# Preparation of γ-Fe_2_O_3_/ZnFe_2_O_4_ nanoparticles by enhancement of surface modification with NaOH

**DOI:** 10.1186/1752-153X-8-40

**Published:** 2014-06-24

**Authors:** Longlong Chen, Jian Li, Yueqiang Lin, Xiaodong Liu, Junming Li, Xiaomin Gong, Decai Li

**Affiliations:** 1 School of Physical Science & Technology, Southwest University, Chongqing 400715, People's Republic of China; 2 School of Mechanical & Control Engineering, Beijing Jiaotong University, Beijing 100044, People's Republic of China

**Keywords:** Nanoparticles, Composite, Surface modification, γ-Fe_2_O_3_, ZnFe_2_O_4_

## Abstract

**Background:**

During liquid-phase synthesis of γ-Fe_2_O_3_ nanoparticles by chemically induced transition in FeCl_2_ solution, enhancement of surface modification by adding ZnCl_2_ was attempted by using NaOH. By using transmission electron microscopy, X-ray diffraction, X-ray photoelectron spectroscopy, energy-dispersive X-ray spectrometry, and vibrating sample magnetometry, the dependence of the synthesis on the amount of additional NaOH was studied.

**Results:**

The experimental results show that the surface of the γ-Fe_2_O_3_ nanoparticles could be modified by adding ZnCl_2_ to form composite nanoparticles with γ-Fe_2_O_3_/ZnFe_2_O_4_ ferrite core coated with Zn(OH)_2_ and adsorbed FeCl_3_, and that modification could be enhanced by adding NaOH.

**Conclusions:**

In the experimental conditions, when the concentration of additional NaOH was below 0.70 M, the amounts of ZnFe_2_O_4_ and Zn(OH)_2_ phases increased slightly and that of adsorbed FeCl_3_ was unchanged. When the concentration of NaOH exceeded 0.70 M, the amount of FeCl_3_, ZnFe_2_O_4_, and Zn(OH)_2_ increased.

## Introduction

Nanoparticles are typically defined as solids that are less than 100 nm in all three dimensions. Many physical phenomena in both organic and inorganic materials have natural length scales between 1 and 100 nm (10^2^ to 10^7^ atoms) [1,2]. A nanocomposite is a material composed of two or more phases with at least one phase with nanometer dimensions. Due to combination of different physical or chemical properties, composite nanoparticles may lead to completely novel materials [3]. For example, the type and geometric arrangement of surface coating on a magnetic core determine the overall size of a nanocomposite colloid and play a significant role in its biological fate in biomedical applications [4].

Magnetic nanoparticles are an important class of functional materials that have attracted increasing interest in terms of their science and their technological applications [5]. Studies on ferromagnetic (FM)–antiferromagnetic (AFM) exchange interactions in systems of fine composite particle have led to interesting applications in improving the performance of permanent magnetic materials or in exceeding the superparamagnetic limit in magnetic recording media [6]. Studies on magnetic nanoparticles have focused on the development of novel technologies for their synthesis. Liquid-phase synthesis is still one of the most commonly used methods to obtain inorganic nanoparticles [7]. Many studies have shown that surface modification, which provides additional functionality to nanoparticles, is easily accomplished after or during synthesis [1]. Recently, we proposed a new method to produce magnetic nanoparticles. This method involves chemically induced transition in which γ-Fe_2_O_3_-based nanoparticles are prepared by processing a precursor based on iron oxide hydroxide and/or metal hydroxide in FeCl_2_ solution [8-10]. In this method, γ-Fe_2_O_3_ nanoparticles can be prepared by using an amorphous coating of FeOOH and Mg(OH)_2_, as described in the following equation [10]:

(1) $$\begin{array}{ll} 2\mathrm{FeOOH} & +\mathrm{Mg}{\left(\mathrm{OH}\right)}_{2}\overset{\mathrm{FeC}l_{2}\;\mathrm{solution}}{\underset{\left(\mathrm{heating}\right)}{\to }}\gamma -Fe_{2}O_{3}↓\\ & +H_{2}O+Mg^{2+}+2OH^{-} \end{array}$$

In another study, surface modification of the particles was undertaken during synthesis by adding ZnCl_2_ to the FeCl_2_ solution to prepare γ-Fe_2_O_3_/ZnFe_2_O_4_ composite nanoparticles [11]. Experimental results show that when the concentration of ZnCl_2_ in solution did not exceed 2 M (50 mL), γ-Fe_2_O_3_/ZnFe_2_O_4_ bioxide nanoparticles coated with FeCl_3_ · 6H_2_O could be prepared. Generally, alkaline solution could assist the precipitation reaction. In the present work, we attempted to enhance the surface modification by adding NaOH to the processing solution. The morphology, crystal structure, surface and bulk chemical composition, and magnetization of the as-prepared products were characterized. The structure of the particles was proposed and the role of NaOH was revealed.

## Experimental

### Preparation

Preparation of the nanoparticles could be divided into two steps. First, the precursor based on amorphous FeOOH and Mg(OH)_2_ was synthesized by coprecipitation of FeCl_3_ and Mg(NO_3_)_2_, as described in detail elsewhere [10]. In the second step, the precursor was added to 400 mL of 0.25 M FeCl_2_ solution, and the resulting mixture was heated to boiling for 20 min. Afterward, a mixture of 50 mL of 1 M ZnCl_2_ solution and 20 mL NaOH solution at a specific concentration was added to the boiling FeCl_2_ solution, and the resulting mixture was boiled continuously for 10 min. Subsequently, the mixture was allowed to cool to room temperature, and the as-prepared particles were allowed to settle. The NaOH concentrations used for the preparation were 0.35, 0.70, 1.40, and 2.10 M, corresponding to the as-prepared samples (1), (2), (3), and (4), respectively. For comparison, modified particles were prepared without adding NaOH (sample (0)).

### Characterization

The morphology of the particles in the samples was observed by transmission electron microscopy (TEM, Philips Tecnai 10), and their crystal structure was analyzed by X-ray diffraction (XRD, XD-2). The chemical species were measured using X-ray photoelectron spectroscopy (XPS, XSAM 800), and energy-dispersive X-ray spectroscopy (EDX, Genesis) equipped in scanning electron microscopy (SEM, Quanta-200). The magnetization was measured by using a vibrating sample magnetometer (VSM, HH-15).

## Results and analysis

**Figure 1:**
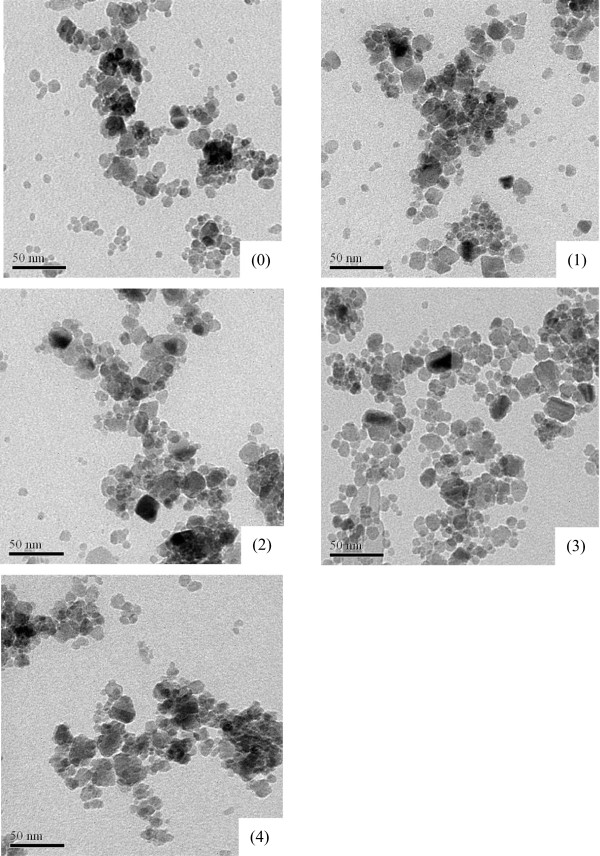
Typical TEM images of the samples prepared without NaOH (0) and with increasing NaOH concentrations: 0.35 (1), 0.70 (2), 1.40 (3) and 2.10 M (4).

TEM images of the samples are shown in Figure 1. It can be seen that sample (0) consisted of nearly spherical nanoparticles. Statistical analysis showed that the size of the particles fit a log-normal distribution, with the median diameter d_g_ about 9.78 nm and the standard deviation lnσ_g_ = 0.28. Samples (1) – (4) images indicated that these samples consisted of irregular flake particles and nearly spherical particles, whose sizes are clearly larger than size of sample (0). This shows that NaOH can stimulate aggregation of the initial particles to grow into larger particles via oriented attachment [12].

**Figure 2:**
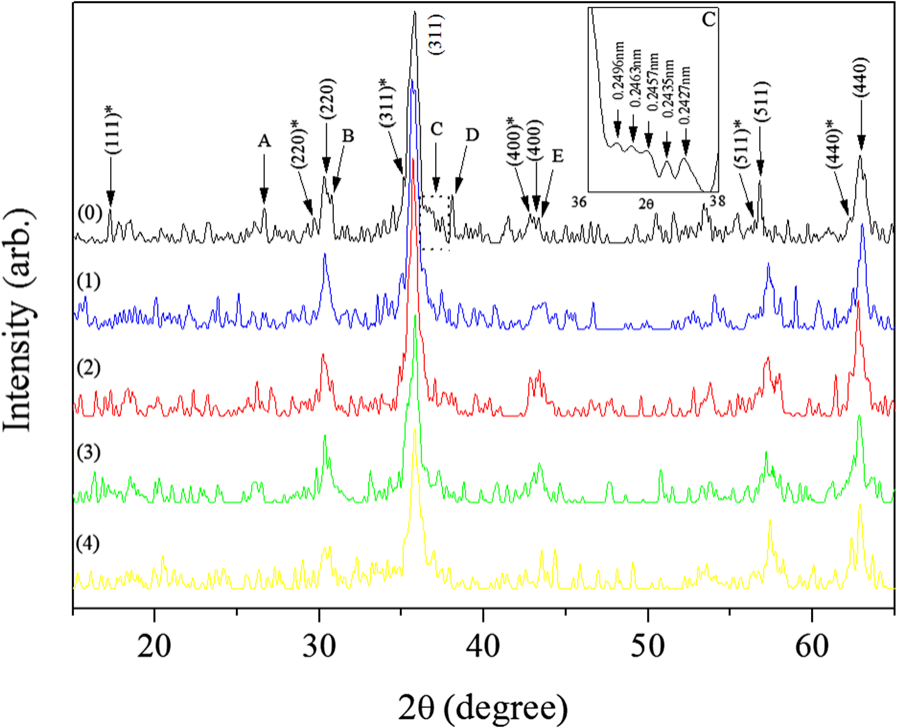
**XRD patterns of the samples prepared without NaOH (0) and with increasing NaOH concentrations: 0.35 (1), 0.70 (2), 1.40 (3) and 2.10 M (4).** Crystallographic indexes (*hkl*), (*hkl*)*, as well as A, B, C, D, and E correspond to the γ-Fe_2_O_3_ (PDF #39-1346), ZnFe_2_O_4_ (PDF #22-1012), and Zn(OH)_2_ (PDF #20-1435) phases, respectively.

XRD patterns (Figure 2) reveal that the samples contained mainly γ-Fe_2_O_3_ and traces of ZnFe_2_O_4_ and Zn(OH)_2_, but no ZnCl_2_.

**Figure 3:**
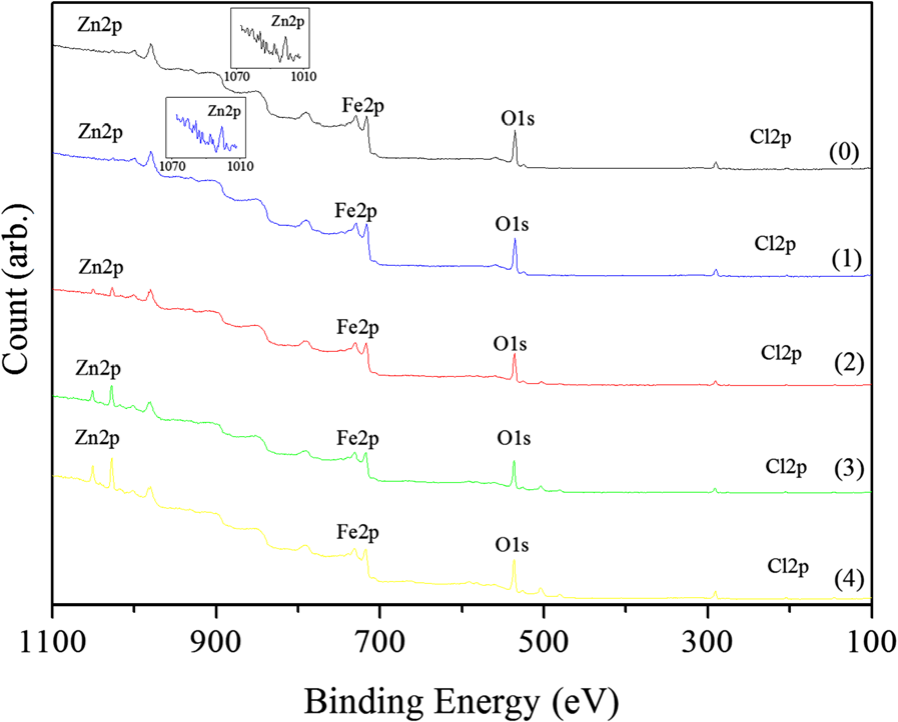
XPS spectra of the samples prepared without NaOH (0) and with increasing NaOH concentrations: 0.35 (1), 0.70 (2), 1.40 (3) and 2.10 M (4).

XPS measurements show that O, Fe, Zn, and Cl but no Mg and Na species were present in the samples, as illustrated in Figure 3. Therefore, the samples consisted of Fe_2_O_3_, ZnFe_2_O_4_, Zn(OH)_2_, and FeCl_3_. Binding energy data are listed in Table 1. As a comparison, the binding energy data of ZnCl_2_[13] are listed also in Table 1. Obviously, the data of measured deviate from data of ZnCl_2_. Quantitative analysis, whose relative error are less that 1 %, shows that relative to sample (0) this ratio of Fe to Cl keeps almost invariant for low NaOH concentrations (up to 0.70 M) – samples (1) and (2), but it increase with NaOH concentrations above that level – sample (3) and (4). Alternatively, to use the same criteria used for the ratio of Fe to Zn of samples, (1) ≈ (2) < (3) < (4). Complete data are listed in Table 2.

**Table 1:** Binding energy data from XPS (eV) for samples prepared without NaOH (0) and with increasing NaOH concentrations: 0.35 (1), 0.70 (2), 1.40 (3) and 2.10 M (4)

	**O1s**	**Fe2p**_**3/2**_	**Zn2p**_**3/2**_	**Cl2p**
0	529.90	710.44	1021.15	198.07
1	529.94	710.71	1021.40	198.19
2	529.93	710.88	1021.17	198.10
3	529.84	710.79	1021.16	198.13
4	530.08	710.98	1021.20	198.28
Fe_2_O_3_^(a)^	530.00	710.70		
ZnFe_2_O_4_^(b)^	530.84	710.95	1020.86	
Zn(OH)_2_^(c)^	531.35		1022.30	
`FeCl_3_^(a)^		711.10		198.70
ZnCl_2_^(d)^			1022.50	199.85

Note: ^(a)^The data are taken from the Hand book of X-ray photoelectron.

^(b)^The data are taken from Ref. [17].

^(c)^The data are taken from Appl. Sur. Sci. 1982, 10: 523, T. L. Barr, J. J. Hackenberg.

^(d)^The data are taken from Ref. [13].

**Table 2:** Atomic percentages of O, Fe, Cl, and Zn from XPS measurements for samples prepared without NaOH (0) and with increasing NaOH concentrations: 0.35 (1), 0.70 (2), 1.40 (3) and 2.10 M (4)

	**O**	**Fe**	**Zn**	**Cl**	**Fe:Zn:Cl**	**Cl:Zn**
0	71.43	25.16	0.61	2.80	1:0.024: 0.111	1:0.22
1	72.36	23.69	1.36	2.59	1:0.057:0.109	1:0.52
2	69.60	25.05	2.65	2.70	1:0.108:0.106	1:1.02
3	68.01	22.89	5.91	3.19	1:0.258:0.139	1:1.86
4	68.06	20.90	7.82	3.22	1:0.374:0.154	1:2.43

The results of EDX measurements of all samples confirm the same chemical species as those detected by XPS. Results of quantitative analysis, whose relative error are less in 2 %, are listed in Table 3. Evidently, the Fe:Cl and Fe:Zn ratios show the same trends for the samples as those observed with XPS.

**Table 3:** Atomic percentages of O, Fe, Cl, and Zn from EDX spectrometry measurements for samples prepared without NaOH (0) and with increasing NaOH concentrations: 0.35 (1), 0.70 (2), 1.40 (3) and 2.10 M (4)

	**O**	**Fe**	**Zn**	**Cl**	**Fe:Zn:Cl**	**Cl:Zn**
0	55.75	42.15	0.48	1.62	1:0.011: 0.038	1:0.29
1	57.85	39.97	0.60	1.58	1:0.015:0.040	1:0.38
2	55.41	41.36	1.70	1.53	1:0.041:0.037	1:1.11
3	55.57	39.63	3.15	1.65	1:0.079:0.042	1:1.88
4	53.56	39.51	4.86	2.07	1:0.123:0.053	1:2.32

**Figure 4:**
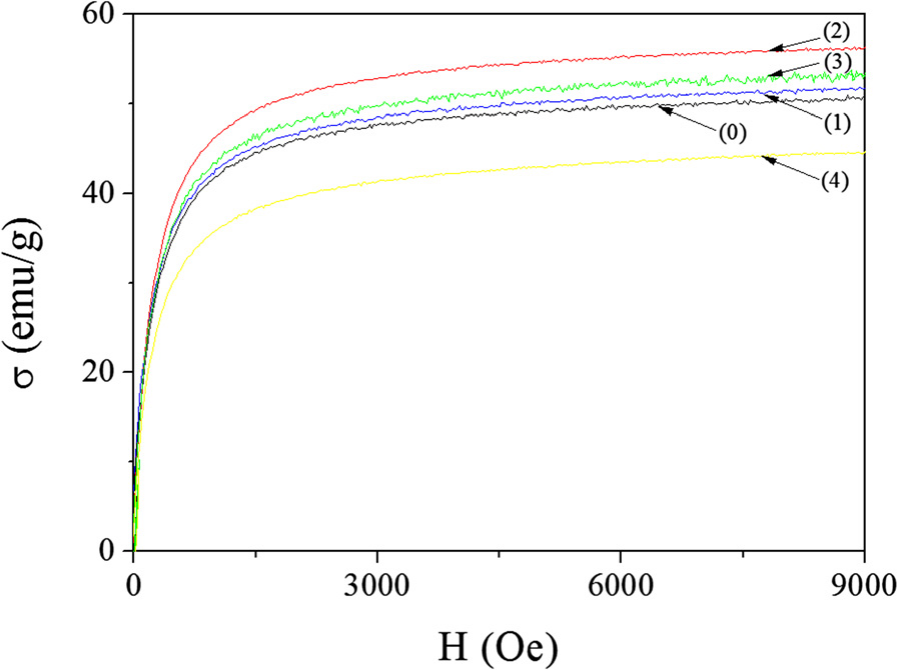
Specific magnetization curves of the samples prepared without NaOH (0) and with increasing NaOH concentrations: 0.35 (1), 0.70 (2), 1.40 (3) and 2.10 M (4).

Figure 4 shows the specific magnetization curves of the samples. All samples showed distinct FM behavior. Their specific saturation values σ_s_ were deduced by plotting σ vs. 1/*H* in the high-field region [14]. These values were found to be 52.29, 53.63, 58.29, 55.09, and 47.15 emu/g for samples (0), (1), (2), (3), and (4), respectively. Magnetization of samples (0) and (1) were nearly the same, that of sample (2) was the strongest, and the magnetization weakened gradually from samples (2) to sample (4).

## Discussion

The experimental results and analysis above indicate that all of the samples were composed of γ-Fe_2_O_3_, ZnFe_2_O_4_, Zn(OH)_2_ and FeCl_3_, and no ZnCl_2_. The experimental results show that the ratios of Fe to Cl and Fe to Zn obtained by XPS were less than those obtained by EDX spectrometry, and the ratio of Cl to Zn obtained by XPS agree with that obtained by EDX spectrometry (see Tables 2 and 3). Since the EDX spectrometry measurements are acquired at micrometer depths whereas XPS data are obtained from the surface layer of nanometer thickness [15,16], the experimental results suggest that the core of the particle is essentially γ-Fe_2_O_3_ and the coating layers are Zn and Cl based.

**Figure 5:**
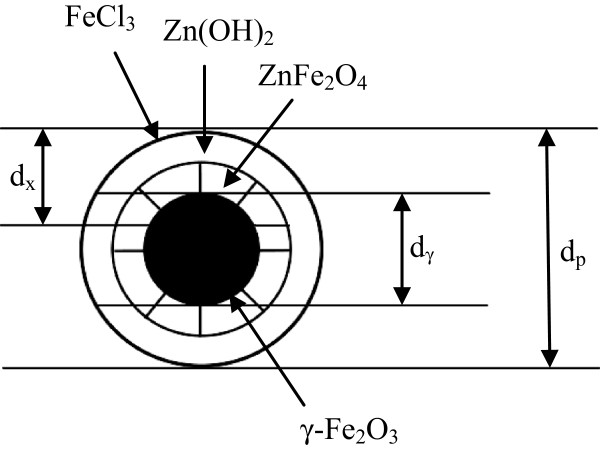
**Schematic diagram of the particle structure.***d*_p_ is the diameter of the composite particle, *d*_γ_ is the diameter of the γ-Fe_2_O_3_ core, and *d*_x_ is the depth detected by XPS.

XRD results show the presence of ZnFe_2_O_4_ and Zn(OH)_2_ phases in addition to the γ-Fe_2_O_3_ phase, but no clear FeCl_3_ crystal phase. These results therefore suggest that the layer of adsorbed FeCl_3_ was very thin and amorphous. Therefore, the structure of the composite nanoparticles consisted of four parts, namely, a γ-Fe_2_O_3_ core, external shells of ZnFe_2_O_4_ and Zn(OH)_2_, and an outermost layer of adsorbed FeCl_3_. A schematic model of this structure is shown in Figure 5. Accordingly, the formation of the composite nanoparticles can be described as follows.

When the FeOOH/Mg(OH)_2_ precursor was thermally treated with FeCl_2_ solution, Mg(OH)_2_ was dissolved, an amorphous FeOOH species was transformed into γ-Fe_2_O_3_ crystallites, and Fe^2+^ was oxidized to Fe^3+^. While adding ZnCl_2_ to the solution, a precipitation reaction took place on the γ-Fe_2_O_3_ crystallites, which can be described as follows:

(2) $$\begin{array}{ll} \;x\left[Fe^{3+}\right) & \left(+3OH^{-}\right]\\ & +y\left[Zn^{2+}+2OH^{-}\right]\to {\left[\mathrm{Fe}{\left(\mathrm{OH}\right)}_{3}\right]}_{x}{\left[\mathrm{Zn}{\left(\mathrm{OH}\right)}_{2}\right]}_{y} \end{array}$$

If *y*/*x* > 1/2, then

(3) $$\begin{array}{ll} \;{\left[\mathrm{Fe}{\left(\mathrm{OH}\right)}_{3}\right]}_{x}{\left[\mathrm{Zn}{\left(\mathrm{OH}\right)}_{2}\right]}_{y}\to & {\left[\mathrm{ZnF}e_{2}O_{4}\right]}_{x/2}{\left[\mathrm{Zn}{\left(\mathrm{OH}\right)}_{2}\right]}_{y-x/2}\\ & +2xH_{2}O \end{array}$$

Thus, ZnFe_2_O_4_ grew epitaxially on the γ-Fe_2_O_3_ crystallites and some Zn(OH)_2_ outside of the ZnFe_2_O_4_ layer was preserved. Clearly, additional NaOH enhanced the reaction so that *x* and *y* increased with increasing NaOH content. In addition, Fe^3+^ and Cl^-^ in the liquid phase were adsorbed and were subsequently converted to composite nanoparticles coated with FeCl_3_. Experimental results show that with increasing NaOH content, the amount of phases based on Zn increased. When the NaOH concentration was lower than 0.70 M, the FeCl_3_ phase was nearly unchanged since the Fe: Cl ratio is almost invariant (Table 2); thus, only when the NaOH concentration exceeded 0.70 M did the amount of FeCl_3_ increase clearly with NaOH concentration due to increasing amount of Cl. This means that the value of *x* in equation (2) increased with NaOH concentration at low NaOH concentrations, i.e., the amount of ZnFe_2_O_4_ phase increased clearly with NaOH concentration when the NaOH concentration did not exceed 0.70 M, but it increased slightly with NaOH concentration when the NaOH concentration was > 0.70 M. As the results in Table 2 show a consistent increase of the Zn: Fe ratio with increasing NaOH concentration, it can be judged that Zn(OH)_2_ increased slightly under low NaOH concentration (<0.70 M) and did clearly under high NaOH consentration (>0.70 M). According to the relation between the specific magnetization and NaOH content, the action of additional NaOH can be discussed further as follows.

The magnetization exhibited stepwise distribution, being strongest in the innermost region of the composite nanoparticle: Zn(OH)_2_/FeCl_3_ < ZnFe_2_O_4_ < γ-Fe_2_O_3_. The specific magnetization of the composite nanoparticle system σ can be described as follows:

(4) $$\begin{array}{ll} \;\sigma = & \phi_{\gamma -\mathrm{Fe}}\sigma_{\gamma -\mathrm{Fe}}+\phi_{\mathrm{Zn}-\mathrm{Fe}}\sigma_{\mathrm{Zn}-\mathrm{Fe}}+\phi_{\mathrm{Zn}}\sigma_{\mathrm{Zn}}\\ & +\phi_{\mathrm{Cl}}\sigma_{\mathrm{Cl}} \end{array}$$

where ϕ_γ-Fe_, ϕ_Zn-Fe_, ϕ_Zn_, and ϕ_Cl_ are mass fractions; σ_γ-Fe_, σ_Zn-Fe_, σ_Zn_, and σ_Cl_ are the specific magnetizations for the γ-Fe_2_O_3_, ZnFe_2_O_4_, Zn(OH)_2_, and FeCl_3_ phases, respectively. σ_γ-Fe_ > σ_Zn-Fe_ > σ_Zn_ (≈ σ_Cl_) since nanoscale ZnFe_2_O_4_ could be weakly ferromagnetic [17], and Zn(OH)_2_ and FeCl_3_ are paramagnetic. Considering as well that ϕ_γ-Fe_ + ϕ_Zn-Fe_ + ϕ_Zn_ + ϕ_Cl_ = 1, equation (4) can be written as

(5) $$\sigma =\sigma_{\gamma -\mathrm{Fe}}-\phi_{\mathrm{Zn}-\mathrm{Fe}}\left(\sigma_{\gamma -\mathrm{Fe}}-\sigma_{\mathrm{Zn}-\mathrm{Fe}}\right)-\phi_{\mathrm{Zn}}\left(\sigma_{\gamma -\mathrm{Fe}}-\sigma_{\mathrm{Zn}}\right)-\phi_{\mathrm{Cl}}\left(\sigma_{\gamma -\mathrm{Fe}}-\sigma_{\mathrm{Cl}}\right)$$

Qualitatively, the variation of the specific magnetization Δσ can be described as follows:

(6) $$\begin{array}{ll} \;\Delta \sigma = & -\Delta \phi_{\mathrm{Zn}-\mathrm{Fe}}\left(\sigma_{\gamma -\mathrm{Fe}}-\sigma_{\mathrm{Zn}-\mathrm{Fe}}\right)-\Delta \phi_{\mathrm{Zn}}\left(\sigma_{\gamma -\mathrm{Fe}}-\sigma_{\mathrm{Zn}}\right)\\ & -\Delta \phi_{\mathrm{Cl}}\left(\sigma_{\gamma -\mathrm{Fe}}-\sigma_{\mathrm{Cl}}\right) \end{array}$$

The mass fraction of every phase should be directly proportional to the molar ratio. The mass fraction is defined as *ϕ*_*i*_ = *m*_*i*_/∑*m*_*i*_, where *m*_i_ represents *m*_γ-Fe_, *m*_Zn-Fe_, *m*_Zn_, and *m*_Cl_, which are the masses of γ-Fe_2_O_3_, ZnFe_2_O_4_, Zn(OH)_2_, and FeCl_3_ phases in the sample, respectively. In the modification to this equation, the mass of the γ-Fe_2_O_3_ phase *m*_γ-Fe_ may be treated as constant. Thus, when *m*_Cl_ was nearly constant under low amounts of additional 0.70 M NaOH, ϕ_Cl_ decreased as *m*_Zn-Fe_ and *m*_Zn_ increased. As a consequence, when ∑ *m*_*i*_ increased at low amounts of NaOH, Δϕ_Cl_ was negative since *m*_Cl_ was unchanged, i.e., -Δϕ_Cl_ > 0; thus, equation (6) can be written as

(7) $$\begin{array}{ll} \;\Delta \sigma = & -\Delta \phi_{\mathrm{Zn}-\mathrm{Fe}}\left(\sigma_{\gamma -\mathrm{Fe}}-\sigma_{\mathrm{Zn}-\mathrm{Fe}}\right)-\Delta \phi_{\mathrm{Zn}}\left(\sigma_{\gamma -\mathrm{Fe}}-\sigma_{\mathrm{Zn}}\right)\\ & +\left|\Delta \phi_{\mathrm{Cl}}\right|\left(\sigma_{\gamma -\mathrm{Fe}}-\sigma_{\mathrm{Cl}}\right) \end{array}$$

Equation (7) shows that increment of the mass fraction of the ZnFe_2_O_4_ and Zn(OH)_2_ phases lowered the value of σ, whereas a decrement in the mass fraction of the FeCl_3_ phase increased it. For sample (1), *x* and *y* in the precipitation reaction described by equation (2) increased slightly compared with those for sample (0); hence, Δϕ_Zn-Fe_, Δϕ_Zn_, and Δϕ_Cl_ were very small compared with their counterparts for sample (0). Therefore, σ of sample (1) was about the same as that of sample (0). For sample (2), *x* and *y* in the precipitation reaction increased, but the increment of *x* could be larger than *y*. Thus, it can be judged from equation (3) that the increment in molar content *x*/2 of the ZnFe_2_O_4_ phase would be larger than that of the Zn(OH)_2_ phase (*y* - *x*/2), i.e., Δϕ_Zn-Fe_ > Δϕ_Zn_. Since σ_γ-Fe_ - σ_Zn-Fe_ < σ_γ-Fe_ - σ_Zn_ (≈ σ_γ-Fe_ - σ_Cl_) and |*Δϕ*_*C*l_| is proportional to Δϕ_γ-Fe_ + Δϕ_Zn_, |*Δϕ*_*C*l_| (σ_γ-Fe_ - σ_Cl_) > Δϕ_Zn-Fe_(σ_γ-Fe_ - σ_Zn-Fe_) + Δϕ_Zn_(σ_γ-Fe_ - σ_Zn_). Consequently, the σ value of sample (2) was greater than those for samples (0) and (1).

When the concentration of additional NaOH exceeded 0.70 M, the amount of FeCl_3_ phase increased with the NaOH content. Therefore, the variation of σ should be described as follows:

(8) $$\begin{array}{ll} \;\Delta \sigma = & -\Delta \phi_{\mathrm{Zn}-\mathrm{Fe}}\left(\sigma_{\gamma -\mathrm{Fe}}-\sigma_{\mathrm{Zn}-\mathrm{Fe}}\right)-\Delta \phi_{\mathrm{Zn}}\left(\sigma_{\gamma -\mathrm{Fe}}-\sigma_{\mathrm{Zn}}\right)\\ & -\Delta \phi_{\mathrm{Cl}}\left(\sigma_{\gamma -\mathrm{Fe}}-\sigma_{\mathrm{Cl}}\right) \end{array}$$

Therefore, the σ weakened in the order of samples (2) to (4).

## Conclusions

During liquid-phase synthesis of γ-Fe_2_O_3_ nanoparticles from precursor composed of amorphous FeOOH and Mg(OH)_2_ by chemically induced transition in FeCl_2_ solution, Mg(OH)_2_ dissolved, FeOOH transformed into γ-Fe_2_O_3_ nanocrystallites, and Fe^2+^ was oxidized partially into Fe^3+^. The surface of the particles could be modified by adding ZnCl_2_ to form γ-Fe_2_O_3_/ZnFe_2_O_4_ composite nanoparticles coated with Zn(OH)_2_ and adsorbed FeCl_3_. Such composite nanoparticles exhibited stepwise distribution of magnetization from inner to outer regions. Thus, they could be easily dispersed in carrier liquid to form excellent ferrofluids [18]. Experimental results indicate that when the amount of ZnCl_2_ solution was constant (1 M, 50 mL), the modification could be enhanced by addition of NaOH. When the concentration of additional NaOH was below 0.70 M, the amount of FeCl_3_ adsorbed was unchanged, but that of ZnFe_2_O_4_ and Zn(OH)_2_ increased slightly, increasing the magnetization of the products. When the concentration of additional NaOH exceeded 0.70 M, the amount of adsorbed FeCl_3_ and ZnFe_2_O_4_ and Zn(OH)_2_ phases increased, and the specific magnetization of the as-prepared products weakened with increasing amount of NaOH. These results show that surface modification during synthesis of the composite nanoparticles γ-Fe_2_O_3_/ZnFe_2_O_4_ coated with Zn(OH)_2_ and FeCl_3_ could be enhanced by additional NaOH to obtain various proportions of phases in the composite particles. This route could be an interesting route for preparing magnetic composite nanoparticles with novel properties. It could potentially be used to prepare other composite nanoparticles based on γ-Fe_2_O_3_. In this regard, it will be investigated further.

## Competing interests

The authors declare that they have no competing interests.

## Authors’ contributions

LC carried out characteristical studies of the nanoparticles, participated in the sequence alignment and drafted the manuscript. JL conceived of the study, and participated in its design and coordination and help to draft the manuscript. YL carried out the preparation of samples. XL carried out the measurements of both VSM and XRD. JL carried out the analysis of both EDX and XPS results. XG performed the analysis of TEM results. DL participated in the design of the study. All authors read and approval the final manuscript.
